# Accelerometer-Assessed Physical Activity in People with Type 2 Diabetes: Accounting for Sleep when Determining Associations with Markers of Health

**DOI:** 10.3390/s23125382

**Published:** 2023-06-07

**Authors:** Alex V. Rowlands, Vincent T. van Hees, Nathan P. Dawkins, Benjamin D. Maylor, Tatiana Plekhanova, Joseph Henson, Charlotte L. Edwardson, Emer M. Brady, Andrew P. Hall, Melanie J. Davies, Thomas Yates

**Affiliations:** 1 Assessment of Movement Behaviours Group (AMBer), Leicester Lifestyle and Health Research Group, Diabetes Research Centre, University of Leicester, Leicester LE5 4PW, UK; 2 National Institute for Health Research, Leicester Biomedical Research Centre, Leicester LE3 9QP, UK; 3 Accelting, 1363 CH Almere, The Netherlands; 4 School of Sport and Wellbeing, Leeds Trinity University, Leeds LS18 5HD, UK; 5 Leicester Lifestyle and Health Research Group, Diabetes Research Centre, University of Leicester, Leicester LE5 4PW, UK; 6 Department of Cardiovascular Sciences, University of Leicester, Leicester LE1 7RH, UK; 7 Hanning Sleep Laboratory and Leicester General Hospital, University Hospitals of Leicester NHS Trust, Leicester LE5 4PW, UK

**Keywords:** intensity gradient, average acceleration, wake, adiposity, physical function, sleep disruption, GENEActiv, GGIR

## Abstract

High physical activity levels during wake are beneficial for health, while high movement levels during sleep are detrimental to health. Our aim was to compare the associations of accelerometer-assessed physical activity and sleep disruption with adiposity and fitness using standardized and individualized wake and sleep windows. People (N = 609) with type 2 diabetes wore an accelerometer for up to 8 days. Waist circumference, body fat percentage, Short Physical Performance Battery (SPPB) test score, sit-to-stands, and resting heart rate were assessed. Physical activity was assessed via the average acceleration and intensity distribution (intensity gradient) over standardized (most active 16 continuous hours (M16h)) and individualized wake windows. Sleep disruption was assessed via the average acceleration over standardized (least active 8 continuous hours (L8h)) and individualized sleep windows. Average acceleration and intensity distribution during the wake window were beneficially associated with adiposity and fitness, while average acceleration during the sleep window was detrimentally associated with adiposity and fitness. Point estimates for the associations were slightly stronger for the standardized than for individualized wake/sleep windows. In conclusion, standardized wake and sleep windows may have stronger associations with health due to capturing variations in sleep durations across individuals, while individualized windows represent a purer measure of wake/sleep behaviors.

## 1. Introduction

Low levels of physical activity are known to be associated with an increased risk of cardiometabolic disease, obesity, and lower fitness [1]. Furthermore, insufficient sleep and/or poor sleep quality are detrimentally associated with a range of health outcomes, e.g., cardiovascular disease risk [2] and cardiometabolic health [3]. Therefore, optimal health is associated with a balanced profile of waking activity and sleep/rest over the 24 h day. Indeed, the latest American Diabetes Association/European Association for the Study of Diabetes (ADA/EASD) consensus guidelines stress the importance of a balance of movement behaviors across the 24 h day to health outcomes associated with type 2 diabetes [4,5]. This includes all movement behaviors that lie along a continuum ranging from limited or no movement to high-intensity activities and include sleep, sitting, and physical activity.

Evidence-based guidelines recommend 7–9 h of sleep per night for adults [6], with both shorter and longer sleep durations associated with poorer health outcomes [7]. Accelerometers are commonly worn 24 h a day to assess movement behaviors, with the average acceleration over the 24 h day often being used as the primary indicator of the physical activity level [8,9]. However, a balanced profile of waking activity and sleep/rest would be reflected in low activity during the recommended sleep period (7–9 h) but higher activity outside of this period.

The importance of this balance was recently demonstrated for the risk of the development of severe COVID-19 [10]. Higher accelerometer-assessed physical activity during the most active continuous two-thirds of the day was associated with lower risk, but higher movement during the least active continuous third of the day (which is indicative of disrupted sleep) was associated with greater risk. The opposing direction of these associations masked the association between physical activity and the risk of COVID-19 when the average acceleration over the 24 h day was used as the physical activity exposure.

It is possible to classify sleep from accelerometer data and thus partition sleep and waking times [11,12]. This enables the direct assessment of sleep disruption and waking physical activity during an individual’s sleep and wake periods, irrespective of the duration of these periods. This contrasts with the consistent time periods for all individuals that are assumed when partitioning data according to the ‘optimal’ balance of two-thirds of the day waking and one-third sleeping. It is important to understand if and where these two approaches agree and disagree when identifying associations with health. It is not always possible to classify sleep accurately, e.g., in low-active populations or those with disturbed sleep [11]; thus, focusing on the most and least active periods of the day may sometimes be a more viable option.

Our aim was to compare the associations of physical activity and sleep disruption with markers of health when physical activity and sleep disruption are assessed on standardized portions of the day with when physical activity and sleep disruption are assessed during accelerometer-determined sleep and wake. For the standardized approach, physical activity was assessed during the most active continuous two-thirds of the day, and sleep disruption was assessed during the least active continuous one-third of the day. For the individualized approach, sleep was classified for each participant [11,12]. Associations with markers of adiposity and fitness that have previously been shown to be associated with accelerometer-assessed activity [13,14] were included.

## 2. Materials and Methods

Participants were from the ongoing cross-sectional, multisite observational study Chronotype of Patients with Type 2 Diabetes and Effect on Glycemic Control (CODEC; clinical trial registry number: NCT02973412). The primary objective of CODEC is to determine if late chronotype is associated with poorer glycemic control compared with early chronotype in a multi-ethnic cohort with established type 2 diabetes. Male and female adults (18–75 years) with established type 2 diabetes (>6 months) were recruited from both primary and secondary care using direct and opportunistic marketing. The inclusion and exclusion criteria for CODEC are as follows:

*Inclusion criteria*

- The participant is willing and able to give informed consent for participation in the study.
- Established T2DM (>6 months since diagnosis).
- Male or female.
- Aged 18 to 75 years inclusive.
- Body mass index (BMI) of less than or equal to 45 kg/m^2^ inclusive.
- No known sleep disorders except obstructive sleep apnea (OSA).
- Glycated hemoglobin (HbA1c) of up to and below 10% (86 mmol/mol).
- Type 1 diabetes.
- Good command of the English language.

*Exclusion criteria*

- The participant is unwilling or unable to give informed consent.
- Anyone without a good command of the English language.
- Anyone of <18 years of age and >75 years of age.
- HbA1c above 10% (86 mmol/mol).
- BMI greater than 45 kg/m^2^.
- A regular cannabis user, that is, weekly use.
- A terminal illness.
- A known sleep disorder that is not OSA.
- Regular use (≥weekly) of medications including those promoting wakefulness, sedatives, melatonin, and medications for nocturnal movement disorders.

All participants provided written informed consent prior to data collection. Ethics approval was obtained from the West Midlands–Black Country Research Ethics Committee (16/WM/0457). CODEC is powered to detect a minimum clinically significant difference in HbA1c of 0.4% between late and early chronotypes and aims to recruit 2247 participants [15]. The study is described in detail elsewhere [15]. The current study was a secondary data analysis, and all participants recruited to date were eligible for inclusion.

Measures extracted from the database included the following: date of birth, sex (male/female), ethnicity (self-reported and collapsed into categories of Caucasian, South Asian (SA), or other, in view of the small number of people from other ethnic groups), diagnosis of OSA, body mass index (BMI, kg·m^−2^), the time since diagnosis of diabetes, diabetes medications, lipid-lowering and blood pressure medications, and the number of additional co-morbidities. The indices of multiple deprivation (IMDs) from self-reported postcodes were used to estimate socio-economic status (SES).

The markers of adiposity were waist circumference (measured to the nearest 0.5 cm) and body fat percentage; the markers of fitness were resting heart rate and physical function (Short Physical Performance Battery (SPPB [16]) and sit-to-stand (STS-60)). Body fat percentage was assessed using bioelectrical impedance (Tanita SC-330ST (Tanita Europe BV, Middlesex, UK)). Resting heart rate was measured after a participant had been seated quietly for 5 min.

The SPPB consists of five chair stands, standing balance, and gait speed.

*Chair stands:* The participant starts from a seated position on a hard, upright chair (such as a dining chair) with their feet flat on the floor and their knees bent at 90 degrees. For the test, the time taken for the participant to stand up fully and then return to sitting without using their hands five times is measured.

*Standing balance:* Tests are conducted in three progressive positions. If the participant is able to complete 10 s in the specified position then the starting position is progressed to the next stage:

- Feet together.
- Semi-tandem.
- Tandem.

*Gait speed:* The time taken for the participant to walk 4 m on a level course is measured. It is measured a second time after a short break.

The SPPB score is the sum of the three tests and can range from 0 to 12 points, with a high score indicating better performance. The STS-60 is similar to the chair stands test in the SPPB but measures how many times the participant can stand from a chair in 60 s. Thus, it is a surrogate measure of muscular endurance that provides data on an additional marker of physical ability [15].

Participants wore GENEActiv accelerometers (ActivInsights Ltd., Cambridgeshire, UK) 24 h a day for up to 8 days on their non-dominant wrists to quantify habitual levels of physical activity and sleep. The monitors were initialized to record accelerations at 100 Hz. Each participant’s device was fitted on the day of their appointment, and they were provided with a prepaid padded envelope in which to return the device and the wake/sleep log at the end of the assessment period.

### 2.1. Accelerometer Processing

Accelerometer files were processed and analyzed with R-package GGIR version 1.11-0 (http://cran.r-project.org; package release date: 4 December 2019) [17]. Signal processing in GGIR includes autocalibration using local gravity as a reference [18]; the detection of sustained abnormally high values; the detection of non-wear; and the calculation of the average magnitude of dynamic acceleration corrected for gravity (Euclidean Norm minus 1 g, ENMO) and averaged over 5 s epochs. All were expressed in milli-gravitational units (mg). Non-wear was imputed using the default setting in GGIR, i.e., invalid data were imputed with the average at similar time-points on different days of the week. Participants were excluded if their accelerometer files showed post-calibration errors of greater than 0.01 g (10 mg) or fewer than 3 days of valid wear (defined as >16 h per day), or if wear data [19] were not present for each 15 min period of the 24 h cycle.

Standardized wake and sleep windows were determined from the most active continuous 16 h (⅔) of the day (M16h) and the least active continuous 8 h (⅓) of the day (L8h), respectively [10]. Individualized wake and sleep windows were calculated using the automated HDCZA sleep detection algorithm [12], with the sleep window defined as starting at sleep onset and ending when waking up after the last sleep episode of the night. The timings of sleep onset and wake were recorded and the mid-points of the sleep windows were determined for the standardized and individualized windows.

The accelerometer outcomes were the average acceleration, relative amplitude (RA), and intensity gradient:

- The **average acceleration** is a proxy for the average intensity of physical activity over a given duration and was calculated over 3 separate time windows for the purposes of this analysis: (1) across the full 24 h day, (2) across the standardized wake and sleep windows, and (3) across the individualized wake and sleep windows. The resulting values were indicative of the overall level of physical activity undertaken within each of the specified time windows [8].
- The **RA** is a composite index of physical activity (average acceleration during the most active continuous 10 h, M10h) and movement during sleep (average acceleration during the least active continuous 5 h, L5h) and is calculated as (M10h − L5h)/(M10h + L5h) [20]. A high RA results from greater waking physical activity and reduced movement during sleep. Scores range from 0 to 1, with higher values indicating a higher amplitude or ‘healthier balance’.
- The **intensity gradient** describes the intensity distribution of physical activity and was calculated over the 24 h day, standardized wake window, and individualized wake window. Specifically, it describes the negative curvilinear relationship between the physical activity intensity and the time accumulated at that intensity [21]. Higher values indicate proportionally more time accumulated in higher-intensity activities or more time spread across the intensity distribution. The intensity gradient is always negative, reflecting the decrease in time accumulated as intensity increases.

### 2.2. Analyses

The data are presented as means (standard deviation (SD)) or medians (inter-quartile range, IQR) for continuous variables and percentages for categorical variables. Paired t-tests were used to assess whether the timings of the standardized wake and sleep windows differed from the individualized sleep and wake windows.

A series of linear regression analyses were used to assess the associations with each health marker for the following:

- The average acceleration over the 24 h day, over standardized wake and sleep windows (mutually adjusted for one another), and over individualized wake and sleep windows (mutually adjusted for one another).
- The RA.
- The intensity distribution of accelerations over 24 h, the standardized wake window, and the individualized wake window.

Accelerometer variables were standardized prior to entry into the models, and the regression coefficient per (SD) was reported for ease of comparison across variables. Regressions were adjusted for the following potential confounders: age, ethnicity, sex, SES, the time since diagnosis of diabetes, and the number of additional comorbidities. Analyses including physical function and heart rate variables were further adjusted for BMI. All analyses were carried out in Stata version 18.0 (StataCorp. LLC, College Station, TX, USA). Statistical significance was set at the alpha level of 0.05.

## 3. Results

CODEC is an ongoing study. To date, accelerometer data are available for 906 participants. Of these, 609 participants had adiposity, fitness, and co-variate data. The descriptive characteristics are presented in Table 1.

**Figure 1:**
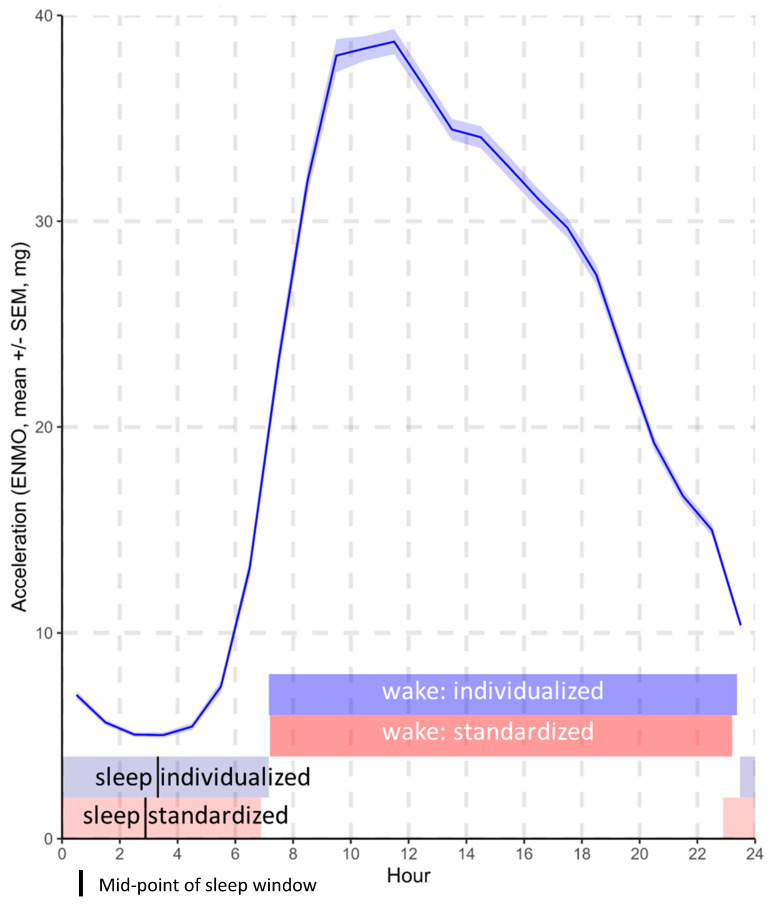
Mean activity by hour of the 24 h day and mean timings of the standardized and individualized wake and sleep windows.

Figure 1 illustrates the average acceleration according to the hour in the 24 h day and the mean timings of the standardized and individualized wake and sleep windows. The accelerometer-determined (individualized) sleep onset was 35 min (95% confidence interval: 30.0, 39.2, and *p* < 0.001) later, and the sleep mid-point was 26 min (22.2, 29.1, *p* < 0.001) later than the onset and mid-point of the standardized sleep window (L8h). The onset timing of the accelerometer-determined (individualized) wake window did not differ from the onset timing of the standardized wake window (M16h).

**Figure 2:**
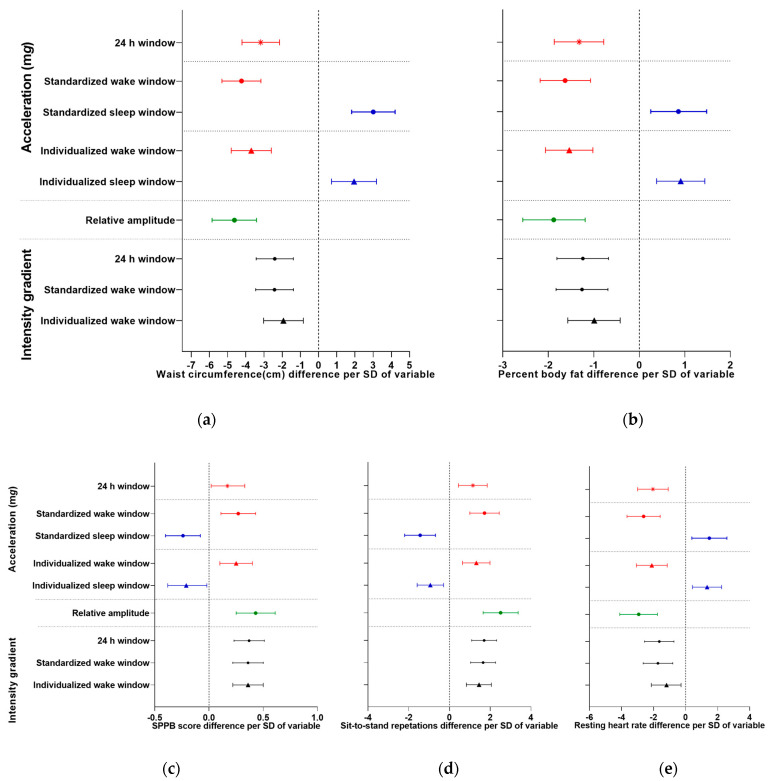
Association between accelerometer outcomes and (**a**) waist circumference, (**b**) body fat percentage, (**c**) Short Physical Performance Battery (SPPB) score, (**d**) sit-to-stand repetitions, and (**e**) resting heart rate over 24 h for standardized wake/sleep windows (M16h/L8h) and individualized wake/sleep windows (accelerometer-determined). Regression coefficients expressed per standard deviation (SD) are reported for ease of comparison across variables. Covariates: age, ethnicity, sex, SES, time since diagnosis of diabetes, and number of additional comorbidities. Analyses with physical function and heart rate variables were further adjusted for BMI. Red = average acceleration during 24 h and wake windows, blue = average acceleration during sleep windows, green = relative amplitude, black = intensity gradient during 24 h and wake windows.

Average acceleration over the 24 h day was beneficially associated with adiposity (−3.2 (−4.2, −2.2) cm waist circumference and −1.3 (−1.9, −0.8) %fat per SD) (Figure 2a,b) and fitness (0.2 (0.0, 0.3) SPPB score per SD, 1.2 (0.4, 1.9) sit-to-stand repetitions per SD, and −2.0 (−3.0, −1.1) bpm resting heart rate per SD) (Figure 2c,d). Considering the partitioned day, the pattern of results was similar, with point estimates for the standardized wake window tending to be stronger and point estimates for the individualized wake window falling between the two.

Average acceleration during the standardized sleep window (L8h) was detrimentally associated with adiposity (3.0 (1.8, 4.2) cm waist circumference and 0.9 (0.3, 1.5) %fat per SD) (Figure 2a,b, Table 2) and fitness (−0.2 (−0.4, −0.1) SPPB score per SD, −1.4 (−2.2, −0.7) sit-to-stand repetitions per SD, and 1.5 (0.4, 2.6) bpm resting heart rate per SD) (Figure 2c–e, Table 2). Except for body fat percentage, point estimates of the associations with acceleration during the individualized sleep window tended to be lower. Associations for the RA tended to be strongest, reflecting the capture of the opposing directions of associations during wake and sleep by this composite variable.

The intensity gradient over the 24 h day was also beneficially associated with adiposity (−2.4 (−3.4, −1.4) cm waist circumference and −1.2 (−1.8, −0.7) %fat per SD) (Figure 2a,b, Table 2) and fitness (0.4 (0.2, 0.5) SPPB score per SD, 1.7 (1.1, 2.3) sit-to-stand repetitions per SD, and −1.6 (−2.6, −0.7) bpm resting heart rate per SD) (Figure 2c–e, Table 2). Associations were similar for the standardized waking window but tended to be slightly lower for the individualized waking window.

## 4. Discussion

The average acceleration and intensity distribution of accelerometer-assessed activity across the 24 h day was beneficially associated with markers of adiposity and fitness in those with type 2 diabetes. A balanced profile was important, whereby higher activity during the waking window was beneficial, but higher movement during the sleep window was detrimental. The pattern of results was similar for the standardized and individualized sleep/wake windows. Associations with the intensity distribution were similar, irrespective of whether a waking or 24 h window was considered.

While associations with the average activity over the 24 h and the standardized waking and individualized waking windows were in the same direction, there were opposing detrimental associations during the sleep window. Previous research suggests that the opposing direction of these associations may, in some cases, mask associations between physical activity assessed over the 24 h window and health and/or risk [10]. This was evident to a lesser extent in this analysis, wherein associations for the standardized waking and individualized waking windows tended to be stronger than those observed for the 24 h day. The stronger associations for the standardized windows may reflect the capture of between-participant variability in sleep duration, which is associated with health [2,3]. While standardized windows attempt to capture wake or sleep behavior, they are influenced by sleep duration. The wake window includes sleep where the sleep duration exceeds 8 h and misses wake where the sleep duration is less than 8 h; conversely, the sleep window includes wake where the sleep duration is less than 8 h and misses sleep where the sleep duration is longer than 8 h.

The relative amplitude uses standardized durations of average activity during wake and sleep to capture the competing directions of these associations in a single metric. However, its composite nature can be difficult to interpret if used in isolation, as it does not provide information on the extent to which associations are due to daytime activity or night-time activity [22]. Considering the opposing impact of activity during sleep/wake windows can provide insight into group differences. For example, Dawkins et al. [23] recently used UK Biobank data to show that relative to white adults, south Asian adults have lower activity but higher sleep/rest disruption.

Focusing on individualized (accelerometer-determined) waking periods led to marginally lower associations with the intensity distribution relative to either the 24 h or the standardized wake window. The 24 h window assesses the distribution of intensity over the wake and sleep periods rolled into one. In contrast, the individualized wake window considers the distribution of intensity over the accelerometer-determined waking period only. The standardized waking window attempts to capture wake behavior but is influenced by sleep duration as outlined above. Thus, the intensity distribution across the standardized wake and 24 h windows may be more strongly associated with health outcomes due to being more sensitive to variations in sleep duration across individuals.

Our results suggest that either standardized or individualized sleep/wake windows can be used to investigate associations between average acceleration and intensity of physical activity and health markers. This offers an alternative approach where classification of sleep can be difficult, e.g., in low-active populations wherein it can be difficult to differentiate between sleep and prolonged periods of inactivity [11].

## 5. Strengths and Limitations

It should be noted that this is a secondary analysis of the CODEC dataset, which was not designed to assess the associations investigated herein. A strength of this analysis was the large sample of adults with type 2 diabetes, in which sleep and physical activity are compromised but important for health [4,5]. However, the CODEC study excludes participants who have a known sleep disorder that is not OSA [15]; thus, patients with type 2 diabetes and a sleep disorder are not represented. The age range of the included sample was broad at 25–75 years, but ~75% of the sample were aged over 60 years. Future research should consider whether these results are generalizable to other populations, for example, children, healthy adults, adults with other chronic diseases, and people with disabilities. It should be noted that in the current study, the mean sleep duration was within the guidelines at just under 8 h, with ~70% of the sample sleeping between 7 and 9 h. It is possible that associations may be more discrepant between standardized and individualized sleep and wake windows in samples with more diverse sleep durations or sleep patterns. Furthermore, capturing the individualized durations of wake and sleep is critical for many research questions, including analyses considering the composition of the day [24], the optimal sleep duration for health [7], and the assessment of adherence to guidelines [6].

## 6. Conclusions

The current data suggest similar associations between the average and distribution of accelerometer-assessed activity and markers of health in adults with type 2 diabetes are evident when standardized and individualized wake and sleep windows are used. Standardized wake and sleep window durations may show stronger associations with health due to capturing variations in sleep duration across individuals, while individualized windows represent a purer measure of the role of waking and sleep behaviors. Where classification of sleep is difficult, using standardized windows offers an alternative approach to accounting for sleep when investigating associations between physical activity and health. Further research should consider whether these results can be generalized to other populations, particularly those who may have more diverse sleep durations and/or sleep patterns.

## Figures and Tables

**Table 1:** Descriptive characteristics.

	Mean ± SD, Median (IQR) *, or Count (%)
Age	66.0 (59.2, 71.0) *
Sex (female)	230 (37.8)
Body mass index (BMI, kg·m^−2^)	30.8 (5.0)
Diagnosis of sleep apnea	46 (7.6)
Years since diabetes diagnosis	8 (4, 14.3) *
Number of diabetes medications	2 (1, 3) *
Medications	
Number on lipid-lowering medication	436 (71.6)
Number on blood pressure medication	398 (65.4)
Number of additional co-morbidities	
0	79 (13.0)
1	171 (28.1)
2	208 (34.2)
3	94 (15.4)
4+	57 (9.4)
*Ethnicity* Caucasian South Asian Other	506 (83.1) 73 (12.0) 30 (4.9)
Index of multiple deprivation †	19,658 ± 9350.1
*Adiposity*	
Fat percentage (%)	34.2 ± 8.9
Waist circumference (cm)	106.2 ± 14.5
*Fitness*	
SPPB score	10.4 ± 1.8
Sit-to-stand reps	22.3 ±7.7
Resting heart rate (bpm)	72.0 ± 11.4
***Accelerometer variables***	
Number of valid days	6.9 ± 0.3
Sleep duration (h:mm)	7:42 ± 1:08
Timings	
*Standardized windows (M16h/L8h)*	
Wake (hh:mm)	07:11 ± 01:15
Sleep onset (hh:mm)	22:54 ± 01:13
Sleep mid-point (hh:mm)	02:54 ± 01:13
*Individualized (accelerometer determined)*	
Wake (hh:mm)	07:11 ± 01:20
Sleep onset (hh:mm)	23:28 ± 01:26
Sleep mid-point (hh:mm)	03:19 ± 01:16
*Average acceleration (mg)*	
24 h	22.4 ± 7.0
*Standardized windows (M16h/L8h)*	
Wake	31.0 ± 10.2
Sleep	5.2 ± 1.5
*Individualized (accelerometer-determined)*	
Wake	30.8 ± 10.2
Sleep	4.5 ± 1.2
*Intensity gradient*	
24 h	−2.726 ± 0.197
Standardized wake	−2.631 ± 0.188
Individualized wake	−2.638 ± 0.205
Relative amplitude	0.94 ± 0.02

N = 609; SPPB: Short Physical Performance Battery; * median (IQR) where not normally distributed; † index of multiple deprivation for self-reported postcode was used to estimate socio-economic status.

**Table 2:** Regression coefficients (expressed per standard deviation (SD) of variable; 95% confidence interval) for association between accelerometer outcomes and waist circumference, body fat percentage, Short Physical Performance Battery (SPPB) score, sit-to-stand repetitions, and resting heart rate over 24 h for standardized wake/sleep windows (M16h/L8h) and individualized wake/sleep windows (accelerometer-determined).

Variable	Window	Regression Coefficient **	95% Confidence Interval	*p*-Value †
**Waist circumference (cm)**				
Average acceleration (mg)	24 h	−3.18	−4.21, −2.15	**<0.001**
	Standardized wake	−4.24	−5.31, −3.17	**<0.001**
	Standardized sleep	3.01	1.82, 4.21	**<0.001**
	Individualized wake	−3.70	−4.80, −2.60	**<0.001**
	Individualized sleep	1.95	0.71, 3.19	**0.002**
Relative amplitude		−4.63	−5.86, −3.41	**<0.001**
Intensity gradient	24 h	−2.41	−3.43, −1.39	**<0.001**
	Standardized wake	−2.42	−3.46, −1.38	**<0.001**
	Individualized wake	−1.93	−3.02, −0.84	**0.001**
**Body fat percentage (%)**				
Average acceleration (mg)	24 h	−1.32	−1.87, −0.78	**<0.001**
	Standardized wake	−1.63	−2.18, −1.07	**<0.001**
	Standardized sleep	0.86	0.25, 1.48	**0.006**
	Individualized wake	−1.54	−2.06, −1.02	**<0.001**
	Individualized sleep	0.91	0.38, 1.44	**0.001**
Relative amplitude		−1.88	−2.56, −1.19	**<0.001**
Intensity gradient	24 h	−1.24	−1.81, −0.68	**<0.001**
	Standardized wake	−1.26	−1.83, −0.69	**<0.001**
	Individualized wake	−0.99	−1.57, −0.42	**0.001**
SPPB * score				
Average acceleration (mg)	24 h	0.17	0.02, 0.33	**0.027**
	Standardized wake	0.27	0.11, 0.43	**0.001**
	Standardized sleep	−0.24	−0.40, −0.08	**0.004**
	Individualized wake	0.25	0.10, 0.40	**0.001**
	Individualized sleep	−0.21	−0.38, −0.02	**0.028**
Relative amplitude		0.43	0.25, 0.61	**<0.001**
Intensity gradient	24 h	0.37	0.23, 0.51	**<0.001**
	Standardized wake	0.36	0.22, 0.50	**<0.001**
	Individualized wake	0.36	0.22, 0.50	**<0.001**
**Sit-to-stand repetitions (per 60 s)**				
Average acceleration (mg)	24 h	1.15	0.44, 1.85	**<0.001**
	Standardized wake	1.72	1.00, 2.44	**<0.001**
	Standardized sleep	−1.44	−2.20, −0.69	**<0.001**
	Individualized wake	1.32	0.64, 1.99	**<0.001**
	Individualized sleep	−0.94	−1.59, −0.29	**0.005**
Relative amplitude		2.51	1.65, 3.37	**<0.001**
Intensity gradient	24 h	1.70	1.09, 2.31	**<0.001**
	Standardized wake	1.65	1.04, 2.26	**<0.001**
	Individualized wake	1.45	0.83, 2.06	**<0.001**
**Resting heart rate (bpm)**				
Average acceleration	24 h	−2.04	−2.99, −1.09	**<0.001**
	Standardized wake	−2.62	−3.66, −1.59	**<0.001**
	Standardized sleep	1.48	0.39, 2.58	**0.008**
	Individualized wake	−2.11	−3.07, −1.15	**<0.001**
	Individualized sleep	1.34	0.43, 2.25	**0.004**
Relative amplitude		−2.93	−4.11, −1.76	**<0.001**
Intensity gradient	24 h	−1.65	−2.56, −0.73	**<0.001**
	Standardized wake	−1.73	−2.65, −0.80	**<0.001**
	Individualized wake	−1.19	−2.10, −0.28	**0.010**

* SPPB: Short Physical Performance Battery; ** regression coefficients expressed per standard deviation (SD) reported for ease of comparison across variables; † significant (*p* < 0.05) values denoted in bold. Covariates: age, ethnicity, sex, SES, time since diagnosis of diabetes, and number of additional comorbidities. Analyses with physical function and heart rate variables were further adjusted for BMI.

## Data Availability

The data that support the findings of this study are not openly available due to containing information that could compromise research participant privacy/consent. Requests for participant-level quantitative data and statistical codes should be made to the corresponding author. Data requests will be put forward to members of the original trial management team who will release data on a case-by-case basis.
